# Capecitabine and Warfarin Interaction: A Case Report With Review of Literature and Management Options

**DOI:** 10.3389/fcvm.2021.707361

**Published:** 2022-01-31

**Authors:** Khalefa Althiab, Manal Aljohani, Sultan Alraddadi, Mohammed Algarni

**Affiliations:** ^1^Department of Pharmacy Service, King Abdulaziz Medical City, Riyadh, Saudi Arabia; ^2^Ministry of National Guard Health Affair, Riyadh, Saudi Arabia; ^3^King Abdullah International Medical Research Center, Riyadh, Saudi Arabia; ^4^King Saud bin Abdulaziz University for Health Sciences, Riyadh, Saudi Arabia; ^5^Adult Oncology Department, King Abdulaziz Medical City, Riyadh, Saudi Arabia

**Keywords:** warfarin, capecitabine, drug interaction, international normalized ratio (INR), case report

## Abstract

Capecitabine is an orally active prodrug of 5-fluorouracil with improved safety and efficacy that is extensively used as an antineoplastic agent. It is converted to 5-Fluorouracil in the liver and tumor tissues. *In vitro* assays did not reveal any significant potential for interaction between capecitabine and its metabolites with warfarin. However, several reports provided clinical evidence of such interaction resulting in an elevated international normalized ratio (INR) and bleeding. Here, we report another case of capecitabine and warfarin concurrent administration that resulted in sub- or supra- therapeutic INR without any bleeding episode or venous-thromboembolic event through the follow-up period. Moreover, a review of available management options is also presented in this paper.

## Introduction

Fluoropyrimidines such as 5-fluorouracil (5-FU) and capecitabine mainstay for several solid malignancies. Capecitabine is approved for colorectal cancer and metastatic breast cancer as mono-therapy and adjuvant therapy (1). Capecitabine is an orally administered prodrug of 5-FU, with almost 100% oral bioavailability, which mimics 5-FU continuous infusion with improved safety and efficacy. Its advantages over 5-FU led to a reduction in the economic burden on both patient and health care systems and infectious complications due to intravenous access. The conversion of capecitabine to 5-FU is catalyzed via thymidine phosphorylase which is found in the liver and several tumors in high levels compared to other healthy tissue (2, 3). Capecitabine is dosed based on the body surface area at 1,250 mg/m^2^ taken twice daily for 14 days, followed by a 7-day rest period over a 21-days cycle (1). On the other hand, warfarin is an oral anticoagulant that antagonizes vitamin K and inhibits the synthesis of clotting factors II, VII, IX, and X in addition to the naturally occurring endogenous anticoagulant proteins C and S. It is indicated for the prevention and treatment of venous thrombosis, pulmonary embolism, atrial fibrillation, myocardial infarction, and prosthetic cardiac valve component embolism (4). Warfarin is a medication with narrow therapeutic index and associated with numerous drug-drug and drug-food interactions, through pharmacodynamic or pharmacokinetic mechanisms (4, 5). It consists of a pair of enantiomers that are extensively and differently metabolized by human cytochrome P450 (CYP 450) isoenzymes. CYP2C9 is the predominant *S*-warfarin enantiomer metabolizing enzyme, while CYP1A2 and CYP3A4 are the major hepatic enzymes contributing to *R*-warfarin enantiomer metabolism (5, 6). Although *S-*warfarin is 5–8 times more potent as a vitamin K antagonist than *R*-warfarin, however, the later has a longer half-life (37.4–88.6 h) compared to that of *S*-warfarin (21.2–42.6 h), and further prolongation of the half-life by decreased metabolism may have greater clinical significance (7). Besides that, *R*-warfarin is a noncompetitive inhibitor of *S-*warfarin's metabolism by CYP2C9, indicating that *S-*warfarin's pharmacokinetic properties may be altered by *R*-warfarin. There is a potential drug-drug interaction between capecitabine and warfarin, alike to that observed between 5-FU and warfarin, speculated to be most likely due to the same mechanism (2, 3, 5). *In vitro* assays of human liver microsome did not reveal any significant potential for interaction between capecitabine or its metabolites and substrates of the CYP 450 isoenzymes 1A2, 2A6, 2C9, 2C19, 2D6, 2E1, or 3A4. However, several post-marketing case reports provided clinical evidence of significant interaction between capecitabine and warfarin, leading to an elevation of international normalized ratio (INR), requiring a black box warning in the package insert. The mechanism of action for the interaction is not well-understood and could be related to down-regulation of CYP2C9 by capecitabine or its metabolites or a pharmacodynamic interaction with warfarin.

## Case Report

A 73-year-old female, new to our institution with a past medical history of traumatic intracranial hemorrhage due to fall, poor mobility, wheelchair-bound, old stroke, cardiomyopathy of undetermined etiology with an ejection fraction of 35–40%, and non-valvular atrial fibrillation (NVAF), which was stable on warfarin 2 mg orally daily for many years, her therapeutic range is 2–3. In January 2020, she was diagnosed with right colonic adenocarcinoma, and later on that month, she underwent a right hemicolectomy. Pathology showed cecal adenocarcinoma moderately differentiated at stage T4N2b. At that time, she was offered adjuvant therapy, but she refused. Few months later, her follow up scans showed liver deposits highly suspicious of metastasis from colonic origin. For colorectal cancer capecitabine is dosed at 1,250 mg/m^2^. However, due to her Eastern Cooperative Oncology Group (ECOG) performance status of three points and taking in account the drug-drug interaction with warfarin, she was started on capecitabine 1000 mg/m^2^, twice daily for 2 weeks, followed by 1 week off. Before her first visit to the Adult Medical Oncology clinic at King Abdulaziz Medical City (KAMC), Riyadh, Saudi Arabia, she already finished three cycles of capecitabine. No other concurrent medications were administered. Her laboratory test showed an elevated INR of 6.98 without any bleeding or bruises. To evaluate the degree of the interaction between capecitabine and warfarin and manage warfarin doses, she was referred to the clinical pharmacist-managed anticoagulation clinic (ATC). Before the referral, the average of all her laboratory results were within normal ranges, including kidney and liver functions. Except for red blood cells and hemoglobin which were 2.5 × 10^∧^12/L and 93 g/L, respectively. The patient interview revealed that before the initiation of capecitabine at a certain point in time, she was shifted from warfarin to rivaroxaban but did not tolerate its gastrointestinal side effects and shifted back to warfarin. Since the patient is on long-term warfarin treatment, the caregivers (patient's daughters) were trained to make “self-manage” adjustment of warfarin doses after performing her coagulation profile testing in a private laboratory. After the capecitabine initiation, the family noticed the patient's INR elevations, reaching up to 5.0 but without any history of minor or major bleeding episodes. They started to monitor the INR every 2 weeks and withholding warfarin on the second day of each cycle and resume it again 1–3 days after the last dose of capecitabine, depending on the INR result. This management resulted in either sub- or supratherapeutic INR. A discussion of the case between the clinical pharmacist and her oncologist, the most responsible physician, resulted in a plan to shift the patient to apixaban after being reviewed by a cardiologist. However, she refused to change warfarin due to her previous gastrointestinal side effects with rivaroxaban. Coagulation profile monitoring was carried out on a weekly basis. The management of warfarin doses on capecitabine period and free period is presented in Table 1. The last recorded INR while writing this paper was 2.45, without any major or minor bleeding episodes or venous-thromboembolic events.

**Table 1:** Warfarin doses with INR trend during capecitabine cycles.

**Management**	**Recorded INR test**		**Warfarin dose (mg/day)**		**Number of days warfarin was withheld**
	**No**.	**Result**	**Capecitabine period (14 days)**	**Capecitabine free period (7 days)**	
Self-managed	1	1.66	2	2	12–15^*^
	2	1.49	2	2	12–15^*^
	3	1.24	2	2	12–15^*^
	4	1.34	2	2	12–15^*^
	5	6.96	2	2	12–15^*^
Anticoagulation clinic	6	2.47	1	2	0
	7	3.80	1	2	2
	8	1.57	0.5	2	0
	9	2.28	0.5	2	0
	10	1.55	1	2	0
	11	2.35	1	2	0
	12	2.21	1	2	0
	13	2.29	1	2	0
	14	2.40	1	2	0
	15	2.28	1	2	0
	16	2.72	1	2	0
	17	2.03	1	2	0
	18	2.75	1	2	0
	19	1.87	1	2	0
	20	2.07	1	2	0
	21	2.43	1	2	0
	22	2.45	1	2	0

* *Number of days withheld self-reported estimate by the patient's caregiver*.

## Discussion

With the increased concomitant use of capecitabine and warfarin, this is another case that confirms the clinical interaction between these two medications. Bleeding events secondary to the interaction have occurred several days to several months after the initiation of capecitabine; these events can occur up to months after the last dose of capecitabine (7, 8). The exact mechanism of this interaction is yet, unknown but may be related to hepatic metabolism. Capecitabine is a prodrug that is converted in a 3-step pathway via thymidine phosphorylase in the liver and the tumor site, releasing its only active metabolite 5-fluorouracil (2, 3). Of importance, gastrointestinal toxicity is an essential common adverse effect of 5-FU that includes nausea, vomiting, and diarrhea. This toxic adverse effect causes cell death of the gastrointestinal epithelium, which could alter warfarin's absorption (7).

Six cases reported concerning the adverse interaction between capecitabine and warfarin are summarized in Table 2. The first two cases in the literature reporting severe coagulopathy with bleeding due to drug-drug interaction between capecitabine and warfarin were of two female patients. The first was a 91-year-old woman diagnosed with adenocarcinoma of the rectum with lung and liver metastases. After 4 cycles of capecitabine, she developed left femoral vein thrombosis, for which she was anticoagulated with warfarin dosed at 2.5 mg/day. After 6 weeks of concurrent administration, the patient was admitted due to vagino-rectal bleeding with an INR of > 10. The second was of a 72-years-old female who had recurrent metastatic breast cancer to the bone. She was controlled on chronic warfarin 2.5 mg/day to treat her pulmonary embolism for 3 years before capecitabine's initiation. After the completion of two cycles, she presented with a 3-day history of loose black stools and INR of > 10 (9). Another two reported cases were of two men with metastatic colon cancer; both were on long-term warfarin with therapeutic INR for a long time before they began capecitabine. Both patients were admitted due to gastrointestinal bleeding in a shocked state, with an INR of > 10. One of these patients was an 81-year-old who was admitted on the fifth day of the first cycle. The second was a 79-year-old who was admitted on the fourth day of the second cycle (10). The fifth case reported was a 67-year-old female who had been well-controlled on long-term warfarin (5 mg/day). After 4.5 weeks of capecitabine initiation for her metastatic breast cancer, she developed hemorrhagic blisters, purpura and ecchymoses, and an INR of 8.56 (11). All of the previously mentioned cases were managed as inpatient settings withholding both medications, administration of vitamin K, and blood products transfusion ± omeprazole infusion. The last case was a 59-year-old female with metastatic breast cancer to bone and lung receiving a chronic mini-dose of warfarin dosed as 1 mg/day as prophylaxis against catheter-associated thrombosis. Before capecitabine initiation, her weekly INR monitoring reports were always below 2. Three weeks after the capecitabine initiation, her INR markedly rose to 8.87 without any signs and symptoms of bleeding. After her coagulation profile was normalized, she was switched to subcutaneous low-molecular-weight heparin (LMWH), and her chemotherapy was restarted without any further consequences (5).

**Table 2:** Summary of reported cases of adverse events between capecitabine and warfarin.

**Patient characteristics**		**Therapy indication**		**Co-administration**			**Bleeding**	
**Case no. (Ref)**	**Age/Gender**	**Warfarin**	**Capecitabine**	**Sequence**	**Duration**	**Reported INR**	**Description**	**Management**
1 (9)	91/F	DVT	Rectal with liver metastases	Capecitabine for 4 cycles before warfarin	6 weeks	>10	Vagino-rectal bleeding	- Holding both agents - IV vitamin K - No further chemotherapy received
2 (9)	72/F	PE	Recurrent metastatic breast cancer	Warfarin for 3 years before Capecitabine	8 weeks	>10	Melena	- Holding both agents - IV hydration and vitamin K - Fresh frozen plasma - Packed red blood cells - No further chemotherapy received
3 (10)	81/M	NR	Metastatic colon cancer	Warfarin before Capecitabine (time interval NR)	1 week	>10	Gastrointestinal in a shocked condition	- Holding both agents - IV vitamin K - Fresh frozen plasma - Packed red blood cells - Omeprazole infusion
4 (10)	79/M	NR	Metastatic colon cancer	Warfarin before Capecitabine (time interval NR)	4 weeks	>10	Gastrointestinal in a shocked condition	- Holding both agents - IV vitamin K - Fresh frozen plasma - Packed red blood cells - Omeprazole infusion
5 (11)	67/F	PE	Metastatic breast cancer	Warfarin for 1 year before Capecitabine	4.5 weeks	8.56	Hemorrhagic blisters, purpura, and ecchymoses	- Holding both agents - IV vitamin K
6 (5)	59/F	CRT prophylaxis	Metastatic breast cancer	Warfarin before Capecitabine (time interval NR)	3 weeks	8.87	No signs and symptoms of bleeding	- Holding both agents - IV vitamin K - Switched to LMWH

*INR, international normalized ratio; DVT, deep vein thrombosis; PE, pulmonary embolism, NR, not reported; CRT, catheter-related thrombosis; LMWH, low-molecular-weight heparin*.

Only two studies addressed the effects of capecitabine and warfarin interaction, as summarized in Table 3. In an observational study, medical records of 69 patients who used capecitabine and warfarin concurrently within 7 days or less of the later use were reviewed. Most patients were diagnosed with breast cancer (49%) or colon cancer (32%). Indications for warfarin use were deep vein thrombosis/pulmonary embolism (*n* = 38), venous access device prophylaxis (*n* = 17), and other indications (*n* = 14). Among the 17 patients who received low-dose warfarin for venous access device prophylaxis, only one bleeding event occurred, and one patient (5.9%) had at least one INR > 3.0. No bleeding events occurred among the 52 patients who received warfarin for indications other than venous access device prophylaxis, although 35 patients (67.3%) had at least one INR > 3.0 and 23 patients (44.2%) had at least one INR > 5.0. Compared with the use of warfarin alone, the study did not find big differences in the rates of bleeding events and elevated INR in patients receiving concomitant capecitabine and warfarin (12). Moreover, a retrospective study of 77 participants analyzed the alerted coagulation profile while on capecitabine with or without warfarin. Tumors were pancreatic or gallbladder (63.6%), colon (23.4%), hepatocellular (5.4%), breast (3.5%), carcinoid and gastric (1.2% each). Liver metastases were present in 32 patients. Only 21 patients were on anticoagulation therapy with warfarin, with an average weekly dose of 18.8 mg, for central-vein thrombosis prophylaxis (48%), deep vein thrombosis (33%), and atrial fibrillation/flutter (19%). Twelve patients were already on warfarin before capecitabine initiation, and nine started warfarin while previously on capecitabine therapy. Eleven patients had an INR > 3 (range, 3.23–11.5); consequently, the incidence of an INR > 3 at 130 days of treatment with capecitabine was 32% with warfarin vs. 4% without warfarin (*P* = 5.1 × 10^−14^). After the discontinuation of capecitabine, INR results returned to their normal ranges. Seven patients developed gastrointestinal bleeding that required hospitalization for aggressive management; four of them were on concurrent administration of capecitabine and warfarin. The incidence of bleeding at 130 days of treatment with capecitabine was 18% with warfarin vs. 2% without (*P* = 4 × 10^−13^). Overall, six patients needed warfarin dose reduction by 1–2.5 mg (13).

**Table 3:** Summary of studies reporting the adverse events between capecitabine and warfarin.

**Study type/number of patients**		**Therapy indication**		**Reported INR**	**Reported bleeding cases**	**Study outcome(s)**
**Type of study (Ref.)**	**No. of patients receiving capecitabine and warfarin concomitantly**	**Warfarin (%)**	**Capecitabine (%)**			
An observational study (12)	69	1. DVT/PE (55.07) 2. Venous access device prophylaxis (24.64) 3. Other (20.29)	1. Breast (49) 2. Colon (32)	−36 patients had at least one INR >3.0 - 23 patients had at least one INR >5.0	One bleeding event in a patient who was on warfarin for venous access device prophylaxis	The study did not find significant differences in the rates of bleeding events and elevated INR in patients receiving concomitant capecitabine and warfarin
A retrospective study ^(15)^	21	1. Central-vein thrombosis prophylaxis (48) 2. DVT (33) 3. AF (19)	1. Pancreatic or gallbladder (63.6) 2. Colon (23.4) 3. HCC (5.4) 4. Breast (3.5) 5. Carcinoid (1.2) 6. Gastric (1.2)	11 patients had an INR >3	GI bleeding was encountered in 7 patients	There is a clinically significant interaction between warfarin and capecitabine

*INR, international normalized ratio; DVT, deep vein thrombosis; PE, pulmonary embolism; AF, atrial fibrillation/flutter; HCC, hepatocellular carcinoma; GI, gastrointestinal*.

### Management

Cancer patients are at higher risk of developing venous thromboembolism (VTE) than non-cancer patients, with an incident of 6.5-fold higher. Thromboembolic event(s) can occur at any time either preceding the diagnosis of cancer, more often, at the time of diagnosis or during treatment resulting in the second leading cause of death among cancer patients. Such population requires comprehensive management, which includes identifying patients that require effectual treatment or pharmacologic prophylaxis to reduce the risk of recurrence (14, 15).

Erratic INR control is seen in patients with cancer making vitamin K antagonists not an option to anticoagulate such population, particularly while receiving chemotherapy. Maintaining therapeutic INR could be challenged by nutritional factors and drug-drug interactions with other concomitant medications, including chemotherapy agents (16). Such management of both agents' concurrent administration is challenging; warfarin dose reduction or switching to an alternative as low-molecular-weight heparin (LMWH) or direct-acting oral anticoagulants (DOACs) are the currently available options. Available anticoagulation options for cancer patients other than warfarin are presented in Table 4.

**Table 4:** Available anticoagulation options for cancer patients (15, 17).

Pharmacologic prophylaxis options		
UFH	5,000 Units every 8 h	
Dalteparin^a,b^	5,000 Units once daily subcutaneous	
Enoxaparin^c^	40 mg once daily subcutaneous	
Fondaparinux^d^	2.5 mg once daily subcutaneous	
Apixaban^e^	2.5 mg orally twice daily	
Rivaroxaban	10 mg orally once daily	
Treatment of established VTE management options		
UFH	80 Units/kg IV bolus followed by 18 Units/kg/h IV*	
Dalteparin^a^^,^^b^^,^^g^	Initially, 200 Units/kg subcutaneous once daily for 30 days	
	Followed by 150 Units/kg subcutaneous once daily	
Enoxaparin^c^	1 mg/kg every 12 hours; or	
	1.5 mg/kg once daily	
Tinzaparin	175 Units/kg once daily subcutaneous	
Fondaparinux^d^	Weight-based dosing regimen	< 50 kg: 5 mg once daily subcutaneous
		50-100 kg: 7.5 mg once daily subcutaneous
		> 100 kg: 10 mg once daily subcutaneous
Apixaban^e^	Initially, 10 mg orally twice daily after that	
	Followed by 5 mg orally twice daily after that	
Rivaroxaban^f^	Initially, 15 mg orally every 12 h for 21 days	
	Followed by 20 mg orally once daily after that	
Edoxaban^h^	Weight-based dosing regimen	≥ 60 mg orally once daily
		≤ 60 kg: 30 mg orally once daily^i^

*aPTT, Activated Partial Thromboplastin; VTE, venous thromboembolism*.

a *FDA approved LMWH for an extended therapy to prevent recurrent thrombosis in patients with cancer*.

b *In renal impairment cancer patients with CrCl ≤ 30 mL/min, monitor anti-factor Xa levels and adjust the dose accordingly to achieve target range 0.5–1.5 international unit*.

c *Mainly renally cleared; avoid in patients with CrCl ≤ 30 mL/min or adjust the dose according to anti-factor Xa levels*.

d *In renal impairment patients with CrCl ≤ 30 mL/min, use is contraindicated by manufacture labeling*.

e *In severe hepatic impairment, Child-Pugh Class C apixaban is not recommended*.

f *Doses to be taken with food*.

g *Maximum daily 18,000 units per day, therapy beyond 6 months not established*.

h *In moderate to severe hepatic impairment (Child-Pugh Class B and C) edoxaban is not recommended*.

i *If the patients' CrCl 30–50 mL/min, or the patient needs concomitant use of a P-glycoprotein inhibitor*.

* *After which adjust the dose based on aPTT*.

### Frequent INR Monitoring and Warfarin Dose Reduction

Generally speaking, more frequent testing is optimal to keep patients within target therapeutic INR, especially during the initial warfarin therapy, if a patient's INR becomes supratherapeutic or subtherapeutic, or if an interacting medication is introduced (18), A case report of a chronically anticoagulated patient with warfarin for a mechanical mitral valve replacement was diagnosed with stage IV metastatic colon cancer. Before initiating capecitabine, therapeutic INR was maintained with an average dose of 10.35 mg/day. While on chemotherapy, the patient was anticoagulated with warfarin; with each cycle, warfarin's dose was adjusted according to the INR result. After completing 3-consecutive cycles of concomitant administration of capecitabine and warfarin, a total reduction of > 85% of the warfarin dose was achieved to maintain therapeutic INR (19). On the other hand, a retrospective study showed that only six out of 21 patients who used capecitabine and warfarin concurrently required warfarin dose reduction by 1–2.5 mg. It is worth mentioning that four of the patients who developed bleeding had an INR of 1.0–1.1 within 4 weeks prior to initiating capecitabine. Only 1 of them developed an elevated INR of 5.9 within 4 weeks after the initiation of capecitabine (13). When managing patients receiving concomitant capecitabine and warfarin, it is crucial to keep in mind the presence of high unpredictable inter-individual variables and the importance of closely monitoring the patients for signs and symptoms of bleeding. Once or twice per week, if applicable, INR monitoring is highly recommended to adjust warfarin's doses accordingly. In our approach, we were targeting to monitor INR twice-weekly; however, we were limited by the patient's age and the complete blood count status that did not allow more frequent INR monitoring (i.e., twice weekly). However, the patient and the caregivers were satisfied with both the monitoring and the INR results.

## Low-Molecular-Weight Heparin

Five clinical trials comparing LMWHs vs. warfarin revealed that they are effective in VTE reduction in cancer patients. Additionally, a meta-analysis of 15 randomized controlled trials confirmed their efficacy. A Cochrane review in 2016 showed a reduction in the risk of symptomatic VTE by roughly half (RR, 0.54; 95%CI, 0.38–0.75) when comparing LMWH prophylaxis with no thromboprophylaxis (20). The absence of drug-drug interaction with capecitabine makes them a considerable option in cancer patients. Also, LMWHs seem to have a more favorable profile in such population. However, several factors may limit their use, including quality of life reduction associated with long-term daily administration of subcutaneous injections, weight-based dosing, the need for dose adjustments in renal impairment, and they are not an option in patients who developed heparin-induced thrombocytopenia (HIT) (14). A study was conducted in patients with active cancer receiving chemotherapy with NVAF to evaluate the safety and efficacy of DOAC vs. LMWH and their associated relevant bleeding-free survival. A total of 302 patients with NVAF and active cancer were included. Among all, 192 (63.5%) were treated with dabigatran, rivaroxaban, apixaban, and edoxaban in 20, 24, 80, and 68 patients, respectively. On the other hand, 110 were treated with LMWH. Systemic embolism and stroke rates were higher in the LMWH group, as reported in seven patients compared to three patients in the DOACs; there were significant differences in relation to major bleeding events (21).

## Direct-Acting Oral Anticoagulants

With the current expansion of treatment and prophylaxis options of VTE, of note, direct-acting oral anticoagulants (DOACs) can be used in cancer patients. Clinical trials of comparing DOACs to warfarin showed that they are non-inferior. The clinical decision of the selected DOACs must be individualized to fit the patients' clinical profile after an in-depth discussion about risks vs. benefits, including patients' risk of bleeding, the presence of any drug-drug interaction with other medications via CYP 3A4 metabolic pathway and P-glycoprotein transport, as well as the patients renal and hepatic functions (14, 15). Although, Clinical data suggest that capecitabine or its metabolites in human may interact mainly with CYP2C9, and to a lesser extent on CYP3A4 or CYP1A2 (22). Up to our knowledge, there is not documented drug-drug interaction nor reported cases between capecitabine or its metabolites and DOACs. Another critical factor to consider, patients receiving chemotherapy frequently suffer from gastrointestinal symptoms, primarily vomiting, diarrhea, and mucositis. Consequently, such patients are at a greater risk of gastrointestinal bleeding and at a higher risk of an altered absorption due to diarrhea episodes that could alter their bioavailability (14). DOACs have been tested extensively in the general population; however, the available data on their safety and efficacy in patients with active cancer and AF remains low. Moreover, the number of cancer patients in the pivotal clinical trials was small; they were mainly excluded from the trial, not to cancer itself, but due to their short life expectancy. Furthermore, cancer-specific information, including the type of cancer, stage, and the concomitant use of chemotherapy, was not collected (16).

## Conclusion

There is clinical evidence of drug-drug interaction resulting in potentiation of coumarin derivatives' effect when co-administered with fluoropyrimidine-based chemotherapy, as reported in several case reports in the literature. Such interaction could occur at any time after the concurrent administration in cancer patients with or without liver metastases. It is reasonable to speculate that such interaction may be due to a similar mechanism as with fluorouracil. This interaction could result from the inhibition of CYP450 2C9 by capecitabine or its metabolites. Awareness of this potentially serious interaction between capecitabine and warfarin will further improve anticoagulation control in cancer patients. Close monitoring of coagulation parameters throughout treatment and to be continued for at least 1 month after the last dose of capecitabine is required in patients receiving both agents concomitantly. Available management options include warfarin dose adjustments, low-molecular-weight heparin, or direct-acting oral anticoagulants.

## Data Availability Statement

The original contributions presented in the study are included in the article/supplementary material, further inquiries can be directed to the corresponding author/s.

## Ethics Statement

Written informed consent was obtained from the individual(s) for the publication of any potentially identifiable images or data included in this article.

## Author Contributions

KA and MAlg were involved in the case management. MAlj and KA wrote the manuscript in consultation with SA and MAlg. All authors contributed to the article and approved the submitted version.

## Conflict of Interest

The authors declare that the research was conducted in the absence of any commercial or financial relationships that could be construed as a potential conflict of interest.

## Publisher's Note

All claims expressed in this article are solely those of the authors and do not necessarily represent those of their affiliated organizations, or those of the publisher, the editors and the reviewers. Any product that may be evaluated in this article, or claim that may be made by its manufacturer, is not guaranteed or endorsed by the publisher.
